# Characterization of RAN Translation and Antisense Transcription in Primary Cell Cultures of Patients with Myotonic Dystrophy Type 1

**DOI:** 10.3390/jcm10235520

**Published:** 2021-11-25

**Authors:** Emma Koehorst, Judit Núñez-Manchón, Alfonsina Ballester-López, Miriam Almendrote, Giuseppe Lucente, Andrea Arbex, Jakub Chojnacki, Rafael P. Vázquez-Manrique, Ana Pilar Gómez-Escribano, Guillem Pintos-Morell, Jaume Coll-Cantí, Alba Ramos-Fransi, Alicia Martínez-Piñeiro, Mònica Suelves, Gisela Nogales-Gadea

**Affiliations:** 1 Neuromuscular and Neuropediatric Research Group, Institut d’Investigació en Ciències de la Salut Germans Trias i Pujol (IGTP), Campus Can Ruti, Universitat Autònoma de Barcelona, 08916 Badalona, Spain; ekoehorst@igtp.cat (E.K.); jnunez@igtp.cat (J.N.-M.); aballester@igtp.cat (A.B.-L.); malmendrote.germanstrias@gencat.cat (M.A.); glucente@igtp.cat (G.L.); aarbex@igtp.cat (A.A.); gpintos@igtp.cat (G.P.-M.); jcoll@igtp.cat (J.C.-C.); aramosf@igtp.cat (A.R.-F.); amartinezp.germanstrias@gencat.cat (A.M.-P.); msuelves@igtp.cat (M.S.); 2 Centre for Biomedical Network Research on Rare Diseases (CIBERER), Instituto de Salud Carlos III, 28029 Madrid, Spain; rafael_vazquez@isslafe.es (R.P.V.-M.); ana_pilar_gomez@iislafe.es (A.P.G.-E.); 3 Neuromuscular Pathology Unit, Neurology Service, Neuroscience Department, Hospital Universitari Germans Trias i Pujol, 08916 Badalona, Spain; 4 IrsiCaixa AIDS Research Institute, 08916 Badalona, Spain; jchojnacki@irsicaixa.es; 5 Laboratory of Molecular, Cellular and Genomic Biomedicine, Instituto de Investigación Sanitaria La Fe, 46026 Valencia, Spain; 6 Joint Unit for Rare Diseases IIS La Fe-CIPF, 46012 Valencia, Spain; 7 Reference Unit for Hereditary Metabolic Disorders (MetabERN), Vall d’Hebron University Hospital, 08035 Barcelona, Spain

**Keywords:** RAN translation, antisense transcription, myotonic dystrophies, primary cell cultures, phenotypic modulators

## Abstract

Myotonic Dystrophy type 1 (DM1) is a muscular dystrophy with a multi-systemic nature. It was one of the first diseases in which repeat associated non-ATG (RAN) translation was described in 2011, but has not been further explored since. In order to enhance our knowledge of RAN translation in DM1, we decided to study the presence of DM1 antisense (DM1-AS) transcripts (the origin of the polyglutamine (polyGln) RAN protein) using RT-PCR and FISH, and that of RAN translation via immunoblotting and immunofluorescence in distinct DM1 primary cell cultures, e.g., myoblasts, skin fibroblasts and lymphoblastoids, from ten patients. DM1-AS transcripts were found in all DM1 cells, with a lower expression in patients compared to controls. Antisense RNA foci were found in the nuclei and cytoplasm of a subset of DM1 cells. The polyGln RAN protein was undetectable in all three cell types with both approaches. Immunoblots revealed a 42 kD polyGln containing protein, which was most likely the TATA-box-binding protein. Immunofluorescence revealed a cytoplasmic aggregate, which co-localized with the Golgi apparatus. Taken together, DM1-AS transcript levels were lower in patients compared to controls and a small portion of the transcripts included the expanded repeat. However, RAN translation was not present in patient-derived DM1 cells, or was in undetectable quantities for the available methods.

## 1. Introduction

Myotonic Dystrophy type 1 (DM1) is an autosomal dominant inherited muscular dystrophy with a multi-systemic nature. Patients display a wide variety of symptoms, including muscle weakness, myotonia, respiratory failure, cardiac conduction defects, cataracts, and endocrine disturbances. In addition, the age of onset varies greatly, from birth up to >70 years. The cause of the variability in clinical manifestation is poorly understood. DM1 is viewed as an RNA gain of function disorder, wherein a CTG expansion in the 3′ untranslated region of the myotonic dystrophy protein kinase (*DMPK*) gene causes the accumulation of expanded transcripts as intranuclear RNA foci, which sequester a number of splicing factors, resulting in loss of function and downstream deregulation of the alternative splicing of several genes [1]. In recent years, the view of DM1 as solely an RNA gain-of-function disorder has changed. Several new discoveries, such as antisense transcription and repeat associated non-ATG (RAN) translation, have added to the complexity of this disease. DM1 antisense (DM1-AS) transcription was first described by Cho and collaborators in 2005 [2]. They reported an antisense transcript, emanating from the adjacent SIX5 regulatory region, which was converted into 21 nt siRNAs. These siRNAs were proposed to have a regulatory role in heterochromatin formation. Huguet and collaborators, however, showed that DM1-AS transcription extends across the CAG repeat, as they could detect DM1-AS RNA after the repeat in the 3′ region of DM1 tissue and they also showed the presence of antisense RNA foci, which did not co-localize with sense RNA foci, in adult mouse models [3]. Similar results were found by Michel and collaborators in human fetal samples [4]. The inclusion of the expanded repeat was confirmed by Gudde and collaborators in 2017, although they found DM1-AS transcripts to be low in abundance and with varying lengths, both including and excluding the CAG repeat [5]. In addition, the DM1-AS strand was found by Zu and collaborators to give rise to RAN-translated polyglutamine stretches [6]. RAN translation is a typical phenomenon seen in repeat expansion disorders, in which peptides are produced from all frames without ATG start codon recognition. Zu and collaborators discovered a novel polyglutamine (polyGln) RAN protein expressed from the antisense CAG expansion transcript of the *DMPK* gene [6]. Nuclear polyGln RAN protein aggregates were found at a low frequency in a DM1 patient’s myoblasts and skeletal muscle (*n* = 1) and at a higher frequency in leukocytes from peripheral blood (*n* = 1) [6]. The nuclear aggregates co-localized with caspase-8, an early indicator of polyGln-induced apoptosis. This suggests that RAN proteins may be an additional mechanism of cytotoxicity in DM1 cells.

Since its first discovery in 2011 by Zu and collaborators, RAN translation has been extensively studied in multiple expansion disorders and great advances have been made [7]. However, the contribution of RAN translation to DM1 pathology has not been further studied since its first report in 2011. Much remains unknown regarding the presence of RAN translation and its mechanism in DM1. Is it equally present across patients, and is its distribution across tissues similar? To what extent does it contribute to the pathology of the disease? In order to further enhance our knowledge of RAN translation in DM1, we decided to study the presence of RAN translation in DM1 primary cell cultures—myoblasts, skin fibroblasts and lymphoblastoids—derived from ten DM1 patients, with a heterogeneous display of subtypes. The RAN-translated polyGln has been described to originate from the antisense strand of the *DMPK* gene. We therefore validated the presence of DM1-AS transcription in our three patient-derived cellular models and lower expression levels were found in patients compared to controls. Additionally, we found that the DM1-AS transcripts were found in both the nucleus and the cytoplasm, of which at least a portion contained the expanded repeat, as shown by the presence of antisense RNA foci in patients. However, the polyGln RAN protein was not present in patient-derived DM1 cells, or was present in such low quantities that it was below the detection limit of the currently available techniques.

## 2. Materials and Methods

### 2.1. Samples

This study was approved by the Ethics Committee of the University Hospital Germans Trias i Pujol and was performed in accordance with the Declaration of Helsinki for Human Research. Written informed consent was obtained from all participants. The study included ten patients with DM1 and thirteen controls with no previous family history of neuromuscular disorders (recruited from the traumatology department, in whom surgery was needed). DM1 diagnosis was confirmed or discarded via triplet-primed PCR in all the study participants. Clinical information of DM1 patients was obtained from medical records and updated at the last visit. We obtained three different samples from eight patients and eleven controls: blood, muscle biopsy, and skin biopsy. The other two patients and two controls only provided a blood sample. All samples were simultaneously obtained from patients with confirmed juvenile, adult or late-onset DM1. The muscle biopsy was obtained from biceps brachii (*n* = 7) or vastus lateralis (*n* = 1) of DM1 patients and from intrinsic forearm or hand muscles of eleven non-DM1 patients. Skin biopsy was obtained with a 0.5 cm skin punch.

### 2.2. Small Pool PCR for Sizing the CTG Repeat

Total genomic DNA was extracted from peripheral blood samples, as previously described [8]. To estimate the length of the expanded mode allele, small-pool PCR (SP-PCR) was carried out with small amounts of input DNA (300 pg), using flanking primers DM-C and DM-DR, as previously described [9,10]. PCR was performed using a Custom PCR Master Mix (Thermo Fisher Scientific, Waltham, MA, USA) supplemented with 69 mM 2-mercaptoethanol, and Taq polymerase (Sigma-Aldrich, Gillingham, UK) at 1 unit per 10 µL. All reactions were supplemented with 5% DMSO and the annealing temperature was 63.5 °C. DNA fragments were resolved by electrophoresis on a 1% agarose gel, and Southern blot hybridized as described in references [9,10]. Autoradiographic images were scanned and the CTG size of the mode was estimated through comparison against the molecular weight ladder using GelAnalyzer 19.1 software (www.gelanalyzer.com, by Istvan Lazar Jr. and Istvan Lazar Sr.).

### 2.3. Cell Culture

Myoblasts were isolated from the biopsied tissue by CD56 magnetic separation. Myoblasts were grown until 60–70% confluency on 0.1% gelatin-coated coverslips in six-well tissue-culture plates in a proliferation medium containing DMEM supplemented with 10% FBS, 22% M-199, 1× PSF, insulin 1.74 μM, l-glutamine 2 mM, FGF 1.39 nM and EGF 0.135 mM. Skin fibroblasts were isolated from biopsied tissue using the explant method. Skin fibroblasts were grown until 70% confluency on 0.5% poly-d-lysin coated coverslips in a six-well tissue culture plate in a proliferation medium containing DMEM, supplemented with 10% FBS and 1× PSF. Peripheral blood mononuclear cells were isolated from blood collected in heparin tubes according to the Ficoll gradient and immortalized using Epstein–Barr virus. Lymphoblastoids were grown until 70% confluency on 0.1% poly-d-lysin coated coverslips in a six-well tissue culture plate in a proliferation medium containing RPMI, supplemented with 10% FBS, 1× PSF and 2 mM l-glutamine. HEK293 cells were cultured until 80% confluency on 0.5% poly-d-lysin coated coverslips in a six-well tissue culture plate in MEM, supplemented with 5% horse serum, 5% FBS, 1% Glutamine and 1% PSF. In addition to the coverslips, cell pellets from each of the cell types were extracted and stored for later use in RNA and protein studies.

### 2.4. cDNA Construct Huntingtin and HEK293 Transfection

The Q17 vector was generously provided to us by R.P.V.-M.. In short, the construct is part of the cDNA of human huntingtin (585 amino acids), containing 17 CAGs, and was cloned into a pcDNA3.1-Gateway. The human huntingtin with the 17 CAGs was in frame with mCherry, a fluorescent. DNA transfections were performed when HEK293 cells reached 80% confluency using Lipofectamine 2000 reagent (Thermo Fisher Scientific, Waltham, MA, USA), according to the manufacturer’s instructions. DNA:lipofectamine ratio was 1:2 and incubation lasted 48 h.

### 2.5. RNA Isolation and Subcellular Fractionation

Total RNA from cultured myoblast, skin fibroblast and lymphoblastoid cell lines was isolated using TRIzol reagent (Thermo Fisher Scientific, Waltham, MA, USA) or the RNeasy plus mini kit (Qiagen, Hilden, Germany), according to the manufacturer’s instructions. Total RNA from a healthy human heart was used as a positive control in all RT-PCR analyses (AM7966, Thermo Fisher Scientific, Waltham, MA, USA).

For RNA isolation from the subcellular fractions, skin fibroblasts were grown to 80% confluency, collected through trypsinization and pelleted through centrifugation at 2000 rpm for 5 min at 4 °C. Cell pellets were washed twice with ice-cold PBS. Cells were resuspended in ice-cold cell disruption buffer (10 mM KCl, 1.5 mM MgCl_2_, 20 mM Tris-Cl (pH 7.5), 1 mM DTT) and incubated on ice for 10 min [11]. Samples were homogenized using a tissue disruptor for 30 s and then Triton X-100 was added to a final concentration of 0.1%. The lysate was centrifuged at 2000 rpm for 5 min at 4 °C, after which the supernatant (cytoplasmic fraction) was removed. Both cytoplasmic and nuclear fraction underwent RNA isolation by the RNeasy plus mini kit (Qiagen, Hilden, Germany), according to the manufacturer’s instructions.

### 2.6. RT-PCR Analysis for DM1-AS Transcript Detection

**Figure 1:**
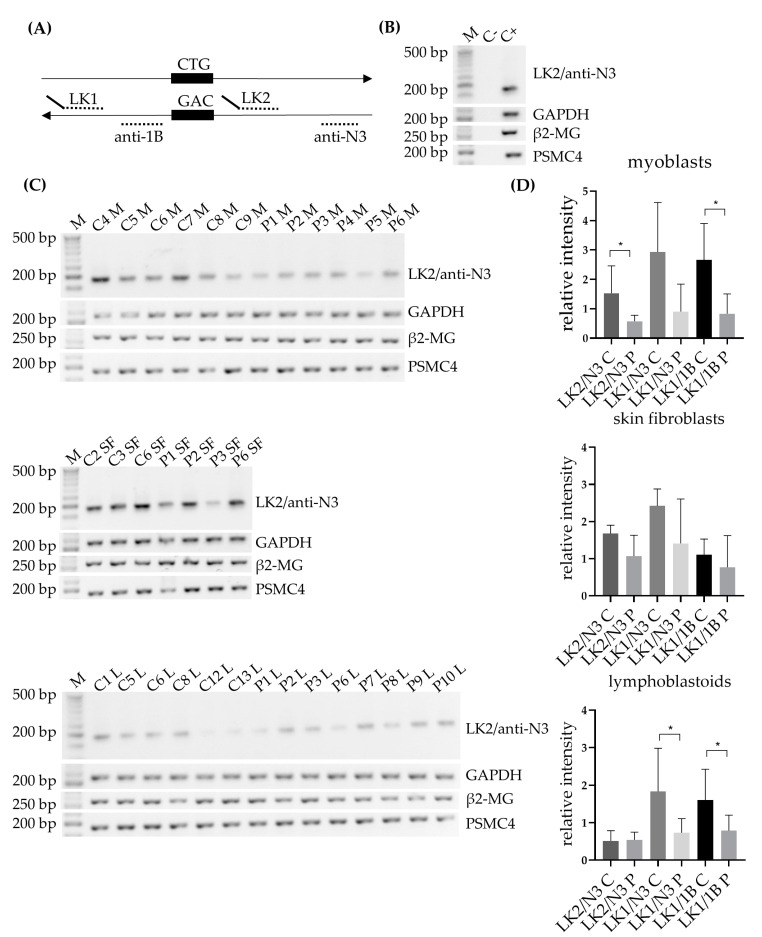
DM1 antisense (DM1-AS) transcripts in DM1 cells. (**A**) Schematic diagram indicating the location of the primers used for the DM1-AS amplification. Both DM1-AS specific primers (LK1 and LK2) have a linker sequence attached, indicated by the tail. Either a linker primer or the strand-specific primers were used for the RT-PCR reactions. A total of three primer combinations were used, LK1 with primers anti-1B/anti-N3 and LK2 with primer anti-N3. (**B**) Positive and negative controls from the RT-PCR reactions; C- = no DNA in the RT-reaction; C+ = control heart RNA as input, the tissue used in the original paper. (**C**) Strand-specific RT-PCRs of the DM1-AS with the DM1-AS specific primers LK2 and anti-N3 in all three primary cell cultures. GAPDH, β2-MG and PSMC4 were used as an endogenous controls. (**D**) Expression of DM1-AS determined by measuring intensity with ImageJ, normalized against the mean of the endogenous controls for all three primer combinations in all three primary cell cultures. Gels of the other two primer combinations can be found in Figure S1. Error bars indicate standard deviations. * = *p*-value below 0.05. Abbreviations: M = marker; C = control; P = DM1 patient; M = myoblasts; SF = skin fibroblasts; L = lymphoblastoids.

For detection of the DM1-AS transcripts, a similar strategy to Zu et al. was followed [6], with some minor changes. In brief, an equivalent of 1 µg total RNA was subjected to cDNA synthesis using SuperScript IV reverse transcriptase (Thermo Fisher Scientific, Waltham, MA, USA) at 55 °C, using random hexamers (50 µM, N8080127, Thermo Fisher Scientific, Waltham, MA, USA). The subsequent PCR was carried out using DM1-AS specific primers LK1, together with anti-N3 or anti-1B, and LK2, together with anti-N3 (Figure 1A; primer details and PCR conditions can be found in Table S1). Both LK1 and LK2 contained a linker sequence, for which a specific primer was created and used in some of the PCR reactions. For endogenous controls GAPDH, β2-microglobulin (β2-MG) and PSMC4 a similar approach was followed (primer details and PCR condition found in Table S1).

To analyze nuclear and cytoplasmic RNA fractions, 150 ng RNA was used for the RT reaction for both fractions. RNA was reverse transcribed using SuperScript IV reverse transcriptase (Thermo Fisher Scientific, Waltham, MA, USA) at 55 °C, using random hexamers (50 µM, N8080127, Thermo Fisher Scientific, Waltham, MA, USA). For RT-PCR analyses, the same approach was used as described above with primer combinations LK1/anti-N3 and LK2/anti-N3. As a nuclear marker, pre-mRNA DMPK was used and GAPDH as an endogenous control (primers and PCR conditions in Table S1).

### 2.7. Immunoblotting

The cell pellets collected from the cell cultures were lysed in a RIPA buffer, supplemented with a cOmplete™ Protease Inhibitor Cocktail (Roche, Basel, Switzerland) and homogenized with a tissue disruptor. After centrifugation, the supernatant was collected and the protein concentration determined with a DC™ Protein Assay Kit II (Bio-Rad Laboratories, Hercules, CA, USA). 20–70 µg of protein was separated on a 3–8% gradient Tris-Acetate gel or an 8% acrylamide gel. Gel proteins were transferred by the iBlot2 system (Thermo Fisher Scientific, Waltham, MA, USA) for Tris-Acetate gels to a nitrocellulose membrane and by wet transfer to a PVDF membrane for the acrylamide gels (Merck, Darmstadt, Germany). Membranes were blocked for one hour in Intercept (TBS) blocking buffer (LI-COR, Lincoln, NE, USA). Immunoblotting was performed with α-DM1 antibody (1:1000, kindly provided by Laura Ranum), 1C2 (clone 5TF1-1C2, 1:250-1:1000, Merck, Darmstadt, Germany), #1874 (clone 3B5H10, Merck, Darmstadt, Germany, 1:1000), TATA-box-binding protein (TBP) (1:250-1:1000, Abcam, Cambridge, UK) or α-tubulin (1:5000, Merck, Darmstadt, Germany) overnight at 4 °C. Appropriate secondary antibodies, anti-rabbit 1:8000 conjugated with IRDye 680RD and anti-mouse 1:8000 conjugated with IRDye 800CW or IRDye 680RD (Thermo Fisher Scientific, Waltham, MA, USA), were used. Band pattern was revealed with an Odyssey Imager (LI-QOR, Lincoln, NE, USA). For favoring the detection of bigger proteins, one of the membranes was stripped with a mild stripping buffer (20 mM glycine, 3 mM SDS, 0.1% Tween 20 in deionized water, with a pH of 2.2), after which the membrane was cut below the 50kD marker and re-blocked and probed with #1874.

### 2.8. Fluorescence In Situ Hybridization (FISH) and Immunofluorescence

Myoblasts, skin fibroblasts and lymphoblastoids grown on coverslips were fixed in 4% paraformaldehyde for 30 min and permeabilized with 0.3% Triton X-100 at 4 °C for 5 min (0.1% Triton X-100 for lymphoblastoids).

For the FISH, coverslips were incubated with 30% formamide in 2× SSC buffer for 30 min at room temperature, followed by an overnight incubation at 37 °C in darkness with a hybridization buffer, containing 0.01 µM Cy3-labelled (CAG)_10_ or (CTG)_10_ probe, 30% formamide, 1% dextran sulfate, 0.02% BSA and 2 mM vanadyl in 2× SSC buffer. The following day, the coverslips were washed with 30% formamide in 2× SSC buffer at 45 °C, 1× SSC buffer at 37 °C and 1× PBS at room temperature, and mounted with ProLong Gold Anti-Fade Mountant with DAPI (Thermo Fisher Scientific, Waltham, MA, USA).

For immunofluorescence, three approaches were followed: a simple immunofluorescence for the detection of RAN-translated polyglutamine protein in all three primary cell cultures, a single-step double immunofluorescence to compare one of the commercial antibodies with the custom antibody used and a two-step double immunofluorescence to assess the co-localization of the found protein with the Golgi apparatus.

For the simple immunofluorescence, coverslips with myoblasts, skin fibroblasts or lymphoblastoids were blocked for 1 h in 5% filtered NGS and incubated overnight at 4 °C with either α-DM1 (1:200, kindly provided by Laura Ranum), 1C2 (Merck, Darmstadt, Germany, ref MAB1574, 1:1000) or #1874 (Merck, Darmstadt, Germany, ref P1874, 1:1000). Next, cells were washed and incubated with goat anti-rabbit conjugated with alexa fluor 488 (Thermo Fisher Scientific, Waltham, MA, USA) for α-DM1 and goat anti-mouse conjugated with alexa fluor 488 (Thermo Fisher Scientific, Waltham, MA, USA) for 1C2 and #1874 for 1 h at room temperature, in darkness. After another round of washes in 1× PBS supplemented with 0.025% Tween-20, coverslips were mounted with ProLong Gold or Diamond Anti-Fade Mountant with DAPI (Thermo Fisher Scientific, Waltham, MA, USA).

For the single-step double immunofluorescence, coverslips were blocked for 1 h in 5% filtered NGS and incubated overnight at 4 °C with two antibodies in the same mix, α-DM1 (1:500 for myoblasts and skin fibroblasts and 1:5000 for lymphoblastoids, kindly provided by Laura Ranum) and 1C2 (1:500, Merck, Darmstadt, Germany). Next, cells were washed and incubated with goat anti-rabbit conjugated with alexa fluor 488 (Thermo Fisher Scientific, Waltham, MA, USA) and goat anti-mouse conjugated with alexa fluor 594 (Thermo Fisher Scientific, Waltham, MA, USA). After another round of washes in 1× PBS, coverslips were mounted with ProLong Gold Anti-Fade Mountant with DAPI (Thermo Fisher Scientific, Waltham, MA, USA).

For the two-step immunofluorescence, coverslips, containing either skin fibroblasts or myoblasts, were blocked for 30 min with the horse blocking solution, containing 5% normal horse serum, 10% normal human serum and 0.02% bovine serum albumin in 1× TBS. Subsequently, the coverslips were incubated overnight at 4 °C with 1C2 (1:200, Merck, Darmstadt, Germany). The following day, the cells were washed three times for 5 min with 1× TBS and incubated at room temperature for 1 h with biotin-labeled horse anti-mouse (1:2000, Thermo Fisher Scientific, Waltham, MA, USA). After another round of washes, the cells were incubated at room temperature for 1 h with streptavidin conjugated with alexa fluor 594 (1:2000, Thermo Fisher Scientific, Waltham, MA, USA). Next, the cells were washed and blocked with the goat blocking solution, containing 5% normal goat serum, 10% normal human serum, 10% Goat anti-horse IgG (Thermo Fisher Scientific, Waltham, MA, USA) and 0.02% bovine serum albumin in 1× TBS. Subsequently, the coverslips were incubated overnight at 4 °C with TGN-38 (1:200, Merck, Darmstadt, Germany). The following day, the cells were washed three times for 5 min with 1× TBS and incubated at room temperature for 1 h with goat anti-mouse conjugated with alexa fluor 488 (1:500, Thermo Fisher Scientific, Waltham, MA, USA). After another round of washes, the cells were mounted with ProLong Gold Anti-Fade Mountant with DAPI (Thermo Fisher Scientific, Waltham, MA, USA).

### 2.9. Image and Statistical Analysis

To assess DM1-AS expression and protein quantity, intensity measurements were taken using ImageJ software, and normalized against the endogenous controls. Differences in transcript expression were calculated using the Mann–Whitney’s non-parametric U test with Graphpad Prism 9.1.2 software; significance level was set at 0.05.

## 3. Results

### 3.1. The DM1 Clinical Phenotype of the Studied Cohort

Our study population consisted of ten DM1 patients, of which the clinical characteristics have been, in part, described previously (Table 1, [12,13]). Eight out of ten DM1 patients provided skin and muscle biopsies, whereas from the other two only lymphoblastoid cell lines were available. The studied DM1 cohort consisted of eight females and two males with an age of onset ranging from 15 to 50 years. Seven individuals were unrelated and three were sisters (P3, P4 and P8). All patients presented with clinical myotonia, but mild muscle impairment was only observed in two patients, reflected by a Biceps MRC of four. Performance in the six-min walking distance test averaged 377 m (range 251–519 m). The muscular impairment rating scale (MIRS) revealed minimal signs of muscular impairment in three of the patients, while five patients showed distal weakness and two patients had mild-moderate proximal weakness. Cardiac problems occurred in all patients, except P3 and P10. Six DM1 patients showed minor ECG alterations, one (P2) a structural cardiopathy (valvulopathy) and P5 had a pacemaker. Five patients needed nocturnal mechanical ventilation, whereas three patients only showed mild changes in the respiratory function test and two patients (P7 and P10) showed no altered respiration. The majority of patients were independent in daily life activities (score of 0–2 on the modified Rankin (mRS) scale), and two patients (P5 and P9) had a moderate to moderately severe disability (scores of 3 and 4, respectively). The average repeat size in blood was 387 CTGs (range 130–619 CTGs) and one patient presented with CCG variant repeats (P7, previously reported [14]).

### 3.2. Lower Expression of DM1-AS Transcripts Found in DM1 Patients Compared to Controls

The RAN-translated polyGln has been described to originate from the antisense strand of the *DMPK* gene. We therefore decided to first validate the presence of DM1-AS transcripts in our three patient-derived primary cell cultures (myoblasts, skin fibroblasts and lymphoblastoids) by using three DM1-AS specific primer combinations, previously described ([6], Figure 1A). The original set-up published by Cho and collaborators and used in the original paper on DM1 RAN translation consisted of using DM1-AS specific primers with a linker sequence attached for strand-specific priming, and a primer complementary to that linker sequence for the subsequent PCRs [2]. However, in our cohort, this setup resulted in an inability to find the DM1-AS transcripts, including our positive control consisting of RNA of a heart control (data not shown), the tissue of origin used in the original paper [6]. When using the DM1-AS specific primers for the subsequent PCRs instead of the linker primer, we detected extreme variability in both DM1-AS transcripts and endogenous controls. We therefore opted for the use of random hexamers for cDNA synthesis and the use of the DM1-AS specific primers for the subsequent PCR. This allowed us to show DM1-AS transcription in all patients and controls with all three primer combinations and with a homogenous housekeeping gene distribution (Figure 1B,C and Figure S1). PCR products were validated by Sanger sequencing. Overall, a lower expression was found in DM1 patients compared to controls with all three primer combinations, which reached significance for LK2/anti-N3 and LK1/anti-1B in myoblasts and LK1/anti-N3 and LK1/anti-1B in lymphoblastoids (Figure 1D). Expression was normalized against each housekeeping gene individually and against the mean of the three endogenous controls. Similar results were obtained with each approach, and the latter was used for the normalization shown in Figure 1. Of note, the LK1/anti-N3 combination, which included the CTG expansion, showed two distinct bands in some of the controls, indicating heterogeneity in their wild-type alleles (Figure S1). This primer combination seemed to favor the wild-type allele, as for the DM1 patients only one patient showed an extra band, which could be the expanded allele, based on the 130 CTG repeat this patient carried (Figure S1A,B).

### 3.3. DM1-AS Transcripts Are Present in the Cytoplasm of DM1 Cells

**Figure 2:**
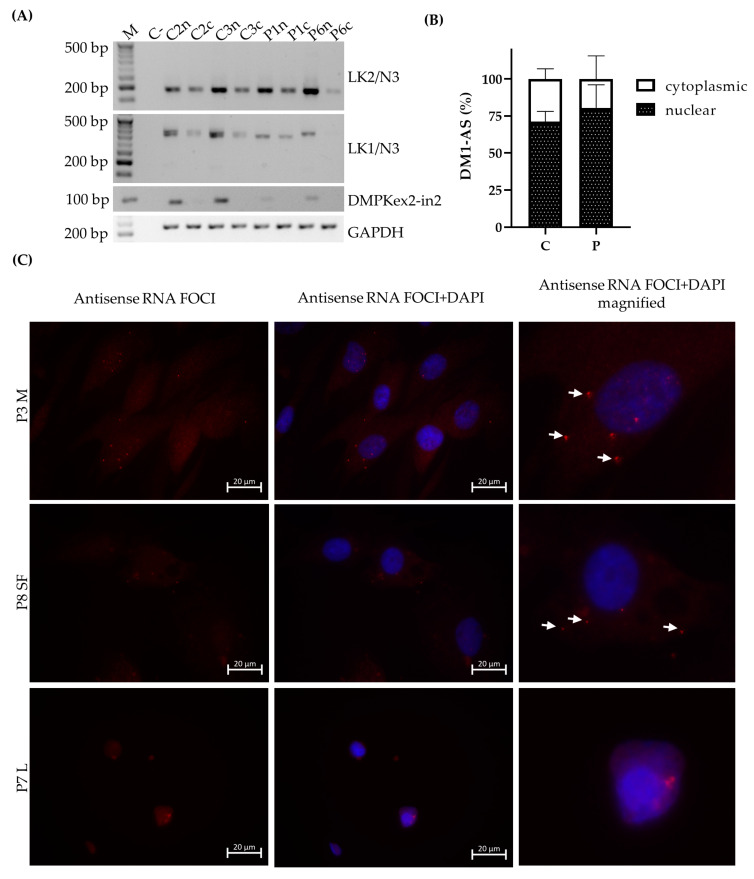
DM1-AS presence in the cytoplasm. (**A**) DM1-AS specific RT-PCR for subcellular fractionated skin fibroblasts with two primer combinations, LK2/anti-N3 and LK1/anti-N3. (**B**) Quantification of DM1-AS signals; bars represent mean +SD. (**C**) Presence of antisense RNA foci in all three cell types of DM1 patients. Cy3-labeled (CTG)_10_ probe (RED) showing antisense RNA foci; nuclei indicated by DAPI (blue) and white arrows indicate cytoplasmic antisense RNA foci. Abbreviations: C = control; P = DM1 patient; *n* = nuclear; c = cytoplasmic; DMPKex2-in 2 = pre-mRNA DMPK as a nuclear marker; GAPDH = endogenous control; M = myoblasts; SF = skin fibroblasts; L = lymphoblastoids.

The results shown above indicate the presence of the DM1-AS transcript in both patients and controls, but for RAN translation to occur, these transcripts need to be able to reach the cytoplasm. To study this, we decided on a dual approach. The first approach was subcellular fractionation of the DM1 cells prior to RNA isolation. We verified the absence of nuclear pre-mRNA *DMPK* in the cytoplasmic fraction to ensure that no nuclear contamination could alter our results. RT-PCRs revealed that, in both patients and controls, the DM1-AS transcripts of two different regions were present in the cytoplasm (Figure 2A). The signal was higher in the nucleus compared to the cytoplasm, and this was slightly more apparent in patients (Figure 2B). In addition, we performed FISH using a Cy3-labeled (CTG)_10_ probe to detect antisense RNA foci and a Cy3-labeled (CAG)_10_ to detect sense RNA foci in DM1 cells. We showed the presence of antisense and sense RNA foci in all DM1 cells (Figure 2C and Figure S2A). Notably, for myoblasts and skin fibroblasts, these antisense RNA foci were also present in the cytoplasm (12.5% of myoblasts and 8.75% of skin fibroblasts). However, this percentage was highly dependent on the patient, as some patients showed only 5% of cells with cytoplasmic antisense RNA foci, whereas others showed 30% (Tables S3 and S5). This variability could not be correlated to, for example, the CTG repeat size. As expected, no sense or antisense RNA foci were found in controls (Figure S2B). Overall, the presence of antisense RNA foci was shown in DM1 cells, although they were less abundant compared to sense RNA foci (Tables S3–S7). Importantly, antisense RNA foci were detected in cytoplasm, indicating that RAN translation is theoretically possible.

### 3.4. RAN Protein Was Undetectable in All Three Primary Cell Cultures

**Figure 3:**
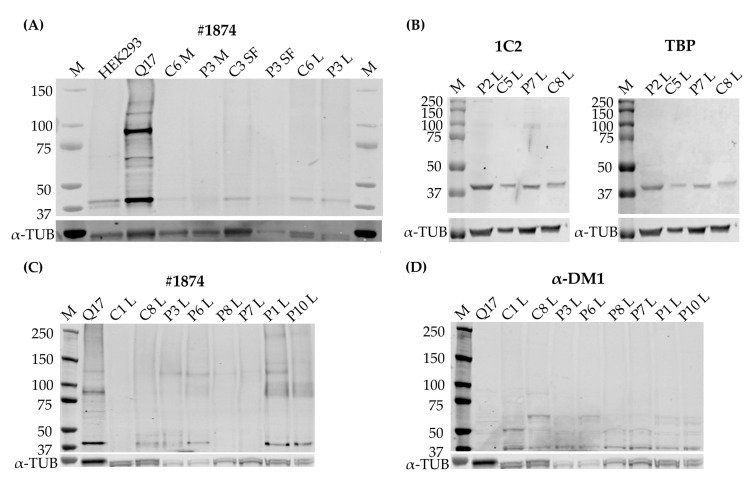
Quantitative analysis of repeat associated non-ATG (RAN) translated polyglutamine (polyGln) protein. (**A**) A representative immunoblot with #1874 1:1000, including all three primary cell cultures, our positive control, a huntingtin vector containing 17 polyglutamine stretches (Q17), and a negative control, consisting of untransfected HEK293 cells (C: *n* = 4, P: *n* = 4). (**B**) Immunoblot showing co-localization of the 42 kD protein found with #1874 and 1C2 with TATA-box-binding protein (TBP) in lymphoblastoids. The antibody shown here is 1C2. (**C**) Immunoblot of lymphoblastoid cell lines, two controls and six patients with #1874 showing several higher bands in certain DM1 patients. (**D**) Immunoblot with the custom antibody α-DM1, showing several non-specific bands, but none that overlap with the bands found with #1874. Abbreviations: M = marker; C = control; P = DM1 patient; HEK293 = untransfected HEK293 cells; Q17 = Q17 huntingtin vector; M = myoblasts; SF = skin fibroblasts; L = lymphoblastoids. #1874 = commercial antibody recognizing polyGln; α-DM1 = custom antibody against the predicted C-terminus of the polyGln RAN protein; α-TUB = α-tubulin as endogenous control.

After validation that the origin of the RAN-translated polyGln was detectable in our primary cell cultures, we moved on to detection of the protein itself. For this, we used three different antibodies, of which two detect polyGln and are commercially available (1C2 and #1874), and one custom antibody, α-DM1, directed against the C-terminus of a predicted glutamine frame of DM1 in the CAG direction (generously provided by Laura Ranum). To make sure our commercial antibodies were able to recognize longer stretches of polyGln, we added two positive controls to our experiments: firstly a huntingtin vector containing 17 glutamines (Q17), in frame with mCherry, transfected into HEK293 cells, with an expected size of approximately 85 kD and secondly a Huntington’s patient lymphoblastoid cell line (Huntingtin expected size: 340–350 kD). The commercially available anti-polyGln antibody #1874 was able to detect the polyGln-containing proteins in the two positive controls, and 1C2 was able to detect the Q17 vector as well (Figure S3A,B), indicating that our approach was able to show polyGln-containing proteins as large as 350 kD. In addition, HEK293 untransfected cells were added as a negative control and only showed a 42 kD protein (Figure 3A and Figure S3B). Protein blots with 1C2 and #1874 in most cases showed a 42 kD protein in both controls and patients in all primary cell cultures, which was also present in both our negative and positive controls (Figure 3A and Figure S3B). This protein was later identified as the TATA-box binding protein (TBP) (Figure 3B). Cutting the membrane above the 42 kD band to favor binding to other proteins did not alter our results (Figure S3C). α-DM1 revealed several different sizes of proteins in all three cell types, ranging from 26 kD to 150 kD, but these were visible in both patients and controls and with no significant differences in intensity (Figure S3D). In addition, the striking observation here was the different size of the polyGln-containing protein that both antibodies bound. Although small, the double staining method clearly showed a different band and the antibodies therefore did not bind the same protein (Figure S3E). The only cellular model to show a band that might correspond to the RAN protein were the lymphoblastoid cell lines, as four out of six patients showed polyGln containing proteins in the upper regions, which were not visible in the controls (Figure 3C). However, these proteins were not recognized by the α-DM1 antibody, which showed several bands in the 37 to 75 kD region in both patients and controls (Figure 3D), and these higher located polyGln-containing proteins could therefore not be validated as a polyGln RAN protein.

**Figure 4:**
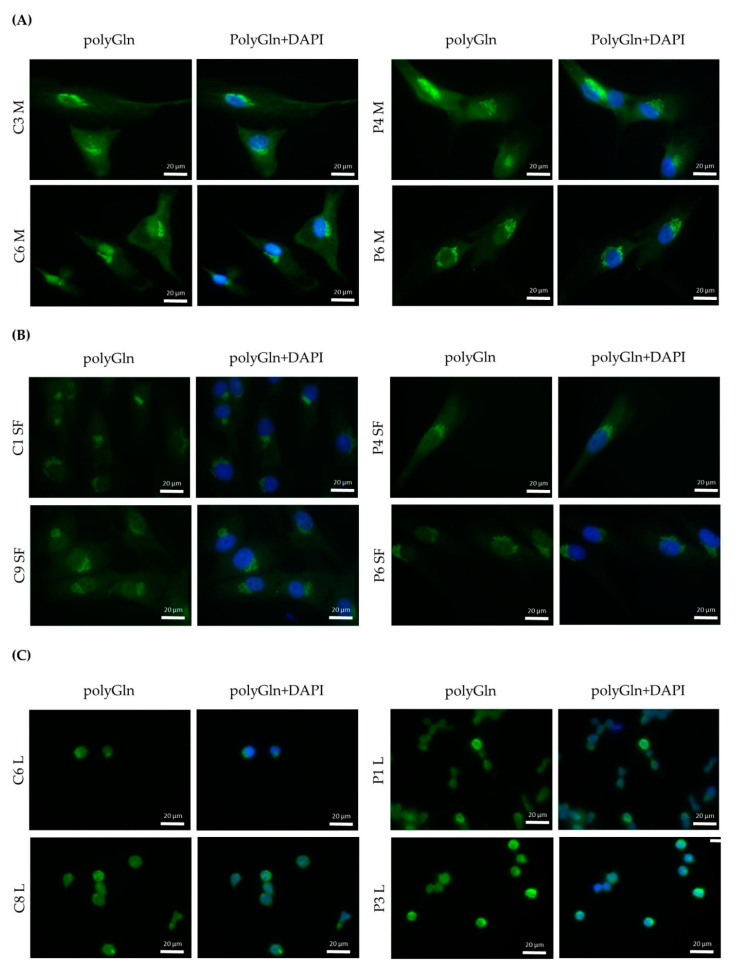
Qualitative analysis of polyGln RAN proteins in all DM1 cells. (**A**) Immunofluorescence polyGln staining with #1874 (alexa fluor-488, green) of human control and DM1 myoblasts. Nuclei indicated by DAPI (blue) (C: *n* = 5, P: *n* = 6) (**B**) Immunofluorescence polyGln staining with #1874 (alexa fluor-488, green) of human control and DM1 skin fibroblasts. Nuclei indicated by DAPI (blue) (C: *n* = 5, P: *n* = 8). (**C**) Immunofluorescence polyGln staining with #1874 (alexa fluor-488, green) of human control and DM1 lymphoblastoids (C: *n* = 4, P: *n* = 5). Nuclei indicated by DAPI (blue). Abbreviations: C = control; P = DM1 patient; polyGln = polyglutamine; M = myoblasts; SF = skin fibroblasts; L = lymphoblastoids.

Although the protein blots did not reveal the polyGln RAN-translated protein, we opted to use a second approach to validate our findings, using immunocytochemistry. By use of the Q17-transfected HEK293 cells we validated the use of our commercial anti-polyGln antibodies in immunocytochemistry (Figure S3F). No differences were observed between patients and controls in all three primary cell cultures (Figure 4). A wide range of concentrations from 1:200 to 1:20,000 was used, but did not alter the original findings (data not shown). Both anti-polyGln antibodies, 1C2 and #1874, showed infrequent staining of the nucleus and an intense aggregate around the nucleus in myoblasts and skin fibroblasts in all cells of both patients and controls (Figure 4A,B, Figures S4 and S5). In lymphoblastoids, due to the limited amount of cytoplasm, it did not show as an aggregate, but rather as an overall intense staining (Figure 4C, Figures S4 and S5). This staining was present in approximately half of the cells, but still visible in both DM1 patients and controls. With the α-DM1, we saw an overall staining of the cytoplasm, with a more intense staining around the nucleus in both DM1 patients and controls and again infrequent staining of the nucleus (Figure S6). In myoblasts, in addition to the intense staining around the nucleus, small bright dots were observed in approximately half of the cells. Again, these were seen in both DM1 patients and controls (Figure S6). This more intense staining was roughly at the same place as the aggregates found with 1C2 and #1874, as illustrated by the double staining (Figure S7).

### 3.5. The Polyglutamine Containing Protein Found Resides in the Golgi Apparatus

**Figure 5:**
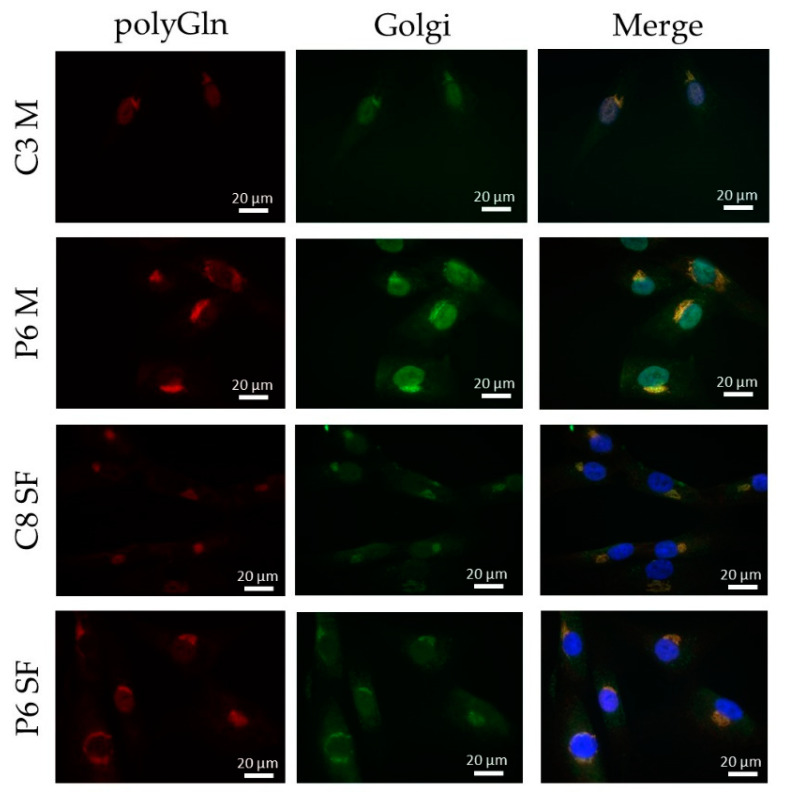
Determination of the origin of the polyGln containing proteins found via the immunofluorescence. Immunofluorescence showing co-localization of the polyGln aggregate found in myoblasts and skin fibroblasts with the commercial antibodies (1C2, alexa fluor-594, red) with the Golgi apparatus (TGN-38, alexa fluor-488 in green). Nuclei stained with DAPI (blue). Abbreviations: C = control; P = DM1 patient; M = myoblasts; SF = skin fibroblasts; polyGln = polyglutamine; Golgi = Golgi apparatus.

Due to the unexpected result of finding a positive staining in both DM1 patients and controls, we decided to further study the origin of this positive staining. The distinct structure found with the 1C2 and #1874 antibody resembled the structure of an organelle and we therefore decided to investigate this hypothesis. A double immunostaining with TGN-38, a marker for the Golgi apparatus, showed an exact match to the structure we found with the 1C2 antibody in DM1 cells (Figure 5).

## 4. Discussion

We studied the presence of antisense transcription and polyGln RAN protein in three primary cell cultures of patients with DM1, namely myoblast, skin fibroblast and lymphoblastoid cell lines, in order to further elucidate its contribution to DM1 pathology.

The presence of antisense transcription, the origin of RAN-translated polyGln, was validated in our three primary cell cultures with three different primer combinations, and lower levels of expression were observed in DM1 patients compared to controls, which reached significance for LK2/anti-N3 and LK1/anti-1B in myoblasts and LK1/anti-N3 and LK1/anti-1B in lymphoblastoids. Of note, the LK1/anti-N3 primer combination, which encompasses the repeat region, revealed that it primarily detected the smaller transcripts, most likely corresponding to the wild-type allele. Only P6 showed an additional band in myoblasts and skin fibroblasts, which, based on the CTG repeat size, could correspond to the expanded repeat. P6 had the smallest expanded repeat (130 repeats) in our cohort and it could be that the other patients, carrying much larger expanded repeats, could not be detected by this method. This could be a potential explanation for the lower levels of expression seen in patients compared to controls. However, the lower levels were also observed with the other two primer combinations that did not encompass the expanded repeat, making it highly unlikely that the lower levels seen were solely due to the binding of the wild-type allele. DM1-AS expression has only been studied by a handful of other groups. Zu and collaborators showed its presence in a heart sample of a DM1 patient and a healthy control; however, the expression patterns were hard to interpret, since an endogenous control was lacking [6]. A study by Gudde and collaborators showed a slightly higher expression in muscle biopsies of DM1 patients when studying RNA-seq data from the myotonic dystrophy deep sequencing data repository [5]. They did, however, mention that globally no obvious differences in read density were observed between DM1 patients and controls. However, when stratified based on inferred MBNL concentration, the most severely affected patients showed a three-fold increase in DM1-AS expression compared to controls, which was in vast contrast to the lower expression levels found in our cohort [5]. Another study, performed by Brouwer and collaborators, showed that in mouse models with increasing CTG repeat length, the DM1-AS transcription levels remained unchanged [15]. Unfortunately, disease severity in Gudde and collaborators’ report was based on the inferred MBNL concentration of DM1 patients, and this was not available for our patients, which meant we could not do a similar stratification. We did, however, have extensive knowledge on the clinical phenotype of our DM1 cohort and had patients from three different clinical subtypes included in our studies, namely juvenile, adult and late-onset. Upon revision, a correlation between expression levels and clinical phenotype could not be found, based on for example, age of onset, muscle involvement (muscle weakness, myotonia) or CTG repeat size, the latter in agreement with the report of Brouwer and collaborators [15]. The sample size of our cohort was rather small for such comparisons or stratification, hindering the analysis. To determine whether DM1-AS transcript expression is linked to disease severity, a bigger cohort is needed.

The presence of DM1-AS transcripts in DM1 cells does not necessarily mean that these transcripts can reach the cytoplasm and be RAN-translated. To further elucidate the localization of these transcripts, cellular fractionation was performed and revealed the presence of DM1-AS transcripts in the cytoplasm of both patients and controls, with a higher percentage in the nuclear fraction. The presence of cytoplasmic DM1-AS transcripts was previously reported by Gudde and collaborators, as they showed the presence of DM1-AS transcripts in the cytoplasmic fraction of myoblasts [5]. However, in both the fractionated and unfractionated DM1-AS pool, it was unclear whether the transcripts possessed the expanded repeat. The LK1/anti-N3 combination already hinted that not all of the DM1-AS transcripts had the expanded repeat. This was also shown by Gudde and collaborators, who found a heterogeneous pool of DM1-AS transcript sizes, with and without the expanded repeat [5]. To determine whether the DM1-AS transcripts in DM1 cells included the expanded repeat, we performed a FISH to detect antisense RNA foci and found that antisense RNA foci were present in both the nucleus and cytoplasm of DM1 cells, indicating that these DM1-AS transcripts contained the expanded repeat and RAN translation was therefore, hypothetically, possible. However, the number of antisense RNA foci compared to sense RNA foci was much lower and they were not present in all cells. In addition, the cytoplasmic antisense RNA foci were even rarer, with only approximately 10% of myoblasts and skin fibroblasts containing cytoplasmic RNA foci, indicating that the presence of DM1-AS transcripts with the expanded repeat in the cytoplasm was quite low. Previous reports on antisense RNA foci in DM1 have also shown that the amount of antisense RNA foci in the nucleus was less compared to sense RNA foci [3,4]. The polyGln RAN protein was undetectable in all three of our primary cell cultures via two different approaches. Western blots revealed a 42 kD polyGln-containing protein with the two commercial anti-polyGln antibodies, which was most likely TBP. In fact, the original immunogen for the 1C2 antibody was the general transcription factor TBP, which contains a 38-Gln stretch and therefore matches our results. It was shown, however, that although TBP will always show up on Western blots in both patients and controls, the antibody favored the binding of longer stretches of polyGln, such as were present in Huntington’s disease and cerebellar ataxia type 1 and 3 [16]. Accordingly, a certain subset of lymphoblastoids did show a band that might correspond to the polyGln RAN protein, but the custom α-DM1 antibody showed a range of non-specific bands in both patients and controls and we were therefore unable to determine the origin of this protein with certainty. In addition, it is difficult to know the exact size of the polyGln RAN protein produced by the DM1-AS, as the disease is prone to somatic mosaicism. This means that cells of the same tissue can carry different CTG expansion sizes and it is therefore also possible to have a range of potential sizes for the protein originating from these transcripts [17,18]. However, Zu and collaborators showed a band just below 60 kD in a patient carrying 85 CTG·CAGs [6]. Our patients carried expansions much larger than that, and when estimating the molecular weight based on the CTG expansion size, it was possible to have polyGln RAN proteins in the range we found within the lymphoblastoid cell lines. This will remain, however, hypothetical, as it seems we do not have a proper functional custom DM1 polyGln RAN antibody and no positive control available to test its functionality.

Immunofluorescence revealed a cytoplasmic aggregate surrounding the nucleus in myoblasts and skin fibroblasts with both commercial anti-polyGln antibodies, which was found to be co-localized with the Golgi apparatus. Since the aggregate was visible in both patients and controls and no apparent differences were seen, this might indicate the detection of another endogenous polyGln-containing protein. For example, ataxin-2, the product of the spinocerebellar ataxia type 2 gene, contains 22 glutamines and resides in the Golgi apparatus [19]. In addition, our immunofluorescence did show staining of the nucleus at high antibody concentrations, which might be due to binding of the transcription factor TBP, also detected by the immunoblots (42 kD band). Taken together, this would mean that both commercial anti-polyGln antibodies bind to several endogenous polyGln-containing proteins, especially at higher antibody concentrations. However, no apparent differences were found between patients and controls across a wide range of concentrations, and the use of α-DM1 antibody did not reveal these similar aggregates. This is in vast contrast to the results previously reported by Zu and collaborators, as they found nuclear polyGln RAN protein aggregates at low frequencies in a DM1 patient’s myoblasts and skeletal muscle (*n* = 1) and at higher frequencies in leukocytes from peripheral blood (*n* = 1) [6]. The 1C2 antibody was used in their experiments to validate the specificity of their custom α-DM1 antibody. Although one of the cell types we used was the same as theirs, i.e., myoblasts, neither antibody was able to find the polyGln RAN protein in our myoblast cell lines, nor in the other two primary cell cultures. In fact, although both types of antibodies showed a protein of approximately 42 kD, our simultaneous staining showed that it was not the same protein, indicating that the antibodies were not able to recognize the same proteins. This was surprising, as the commercial antibody was used to validate the custom antibody in the paper of Zu and collaborators [6]. Our DM1-AS results suggested that the presence of DM1-AS transcripts containing the expanded repeat in the cytoplasm of DM1 cells is quite a rare occurrence. This highly affects the chance of producing polyGln RAN proteins. In addition, polyGln-containing proteins are very common in healthy subjects. Taking these two notions together, it might be plausible that with current techniques, sensitivity is too low to detect such low quantities of the polyGln RAN protein, which in addition is hindered by the presence of other polyGln containing proteins.

Although we were unable to detect polyGln RAN proteins in our DM1 cells, much progress has been made in other repeat expansion disorders displaying RAN translation, which could help in the field of DM1. Nine expansion disorders have been added since the first discovery of RAN translation in SCA8 and DM1: C9orf72 amyotrophic lateral sclerosis/frontotemporal dementia [20,21,22], fragile X tremor/ataxia syndrome [23], Huntington’s disease (HD) [24,25], spinocerebellar ataxia 3 and 31 [26,27], Fuchs’ endothelial corneal dystrophy [28] and myotonic dystrophy type 2 (DM2) [29]. Of these, SCA8, SCA3 and HD are the three repeat expansion disorders in which the RAN proteins originate from a CAG expansion, and can therefore result in polyGln RAN proteins. Interestingly, in vivo, none of these diseases show polyGln RAN proteins, but instead produce poly-alanine, and for HD additionally poly-serine RAN proteins. It might be interesting to include custom antibodies for the two additional homo-polymeric protein possibilities with regard to DM1. Although the name suggests a close relationship between DM1 and DM2, the underlying expansion in DM2 is a CCTG expansion and therefore results in complex poly-LPAC (sense) or poly-QAGR (antisense) RAN proteins, and is thus not hindered by the presence of endogenous polyGln proteins. The study was performed in autopsy brains, a tissue not yet studied for DM1, which might also be worth exploring.

In conclusion, DM1-AS transcript levels were lower in patients compared to controls and were present in both the nucleus and the cytoplasm of DM1 cells. Only a small portion of the DM1-AS transcripts contained the expanded repeat, substantially lowering the possibility of RAN translation in DM1. The polyGln RAN protein was not present in patient-derived DM1 cells, or was present in such low quantities that it is below the detection limit of the currently available techniques.

## Figures and Tables

**Table 1:** Clinical characteristics of Myotonic Dystrophy type 1 (DM1) patients.

Patient	Sex	Age of Onset (y)	Age at Sampling (y)	Biceps MRC	Myotonia (s)	6-MWD (m)	MIRS	mRS	Cataracts	Cardiopathy	Spirometry	Repeat Size (CTGs)
P1	F	15 *	36	4	0.52	348	4	2	no	LAFB	Altered PFT	445
P2	M	48	54	5	0.67	251	3	2	yes	Valvulopathy	NMV	381
P3	F	36	41	5	0.73	368	2	1	yes	none	NMV	338
P4	F	42	46	5	0.98	338	3	1	yes	1st-degree AV block	NMV	246
P5	F	27	40	4	NP	NP	4	4	yes	Pacemaker	Altered PFT	374
P6	M	36	41	5	0.96	519	3	2	no	LAFB	Altered PFT	130
P7	F	50	62	5	NP	436	2	1	yes	1st-degree AV block	none	561
P8	F	35	38	5	NP	NP	3	2	no	1st-degree AV block	NMV	619
P9	F	30	51	5	NP	NP	4	3	yes	1st-degree AV block	NMV	NP
P10	F	18	34	5	NP	NP	3	2	yes	none	none	NP

F = female; M = male; MRC = Medical Research Council; NP = not performed; 6MWD = 6-min walking test; AV = atrioventricular; LAFB = left anterior fascicular block; mRS = modified Rankin Scale; MIRS = Muscular Impairment Rating Scale; NMV = nocturnal mechanical ventilation; PFT = pulmonary function test; * Although the exact age of disease onset was impossible to determine, based on the symptoms displayed at first visit (age 36), which commonly appear with an early onset (including oval pallor and temporal atrophy), disease onset was considered to have been during adolescence.

## Data Availability

The data presented in this study are available on request from the corresponding author.

## Supplementary Materials

The following are available online at https://www.mdpi.com/article/10.3390/jcm10235520/s1. Table S1: Primer sequences. Figure S1: DM1-AS transcripts in DM1 cells of the additional two primer combinations studied. Figure S2: RNA foci overview. Table S2: Sense RNA foci details in DM1 myoblasts. Table S3: Antisense RNA foci details in DM1 myoblasts. Table S4: Sense RNA foci details in DM1 skin fibroblasts. Table S5: Antisense RNA foci details in DM1 skin fibroblasts. Table S6: Sense RNA foci details in DM1 lymphoblastoids. Table S7: Antisense RNA foci details in DM1 lymphoblastoids. Figure S3: Quantitative analysis of the polyGln RAN protein and antibody validation. Figure S4: Qualitative analysis of polyGln RAN proteins with the #1874 antibody of additional samples. Figure S5: Qualitative analysis of polyGln RAN proteins with the 1C2 antibody. Figure S6: Qualitative analysis of polyGln RAN proteins with the α-DM1 antibody. Figure S7: Double immunofluorescence with the α-DM1 antibody and 1C2 antibody.
