# Supplementary material for: Characterization of RAN Translation and Antisense Transcription in Primary Cell Cultures of Patients with Myotonic Dystrophy Type 1
Source: J Clin Med. 2021 Nov 25;10(23):5520. doi: 10.3390/jcm10235520 (PMC8658563; doi:10.3390/jcm10235520)
Supplement: Supplementary file 1 [file jcm-10-05520-s001.zip › jcm-1465039-supplementary.pdf]

Supplementary Materials

Table S1. Primer sequences.

Name primer	Sequence	Tm	Cycles	Expected size
LK1	5′-CGCCTGCCAGTTCACAACCGCTCCGAGCGT-3′			
LK2	5′-GACCATTTCTTTCTTTCGGCCAGGCTGAGGC-3′			
linker	5′-CGACTGGAGCACGAGGACACTGA-3′			
Anti-N3	5′-GAGCAGGGCGTCATGCACAAG-3′	63	30	LK1: 349 bp<br>LK2: 162 bp
Anti-1B	5′-GCAGCATTCCCGGCTACAAGGACCCTTC-3′	67	30	LK1: 150 bp
GAPDH-fw	5′-GAAGGTGAAGGTCGGAGTC-3′			
GAPDH-rev	5′-GAAGATGGTGATGGGATTTC-3′	58	30	226 bp
β2-MG-fw	5′-CCAGCAGAGAATGGAAAGTC-3′			
β2-MG-rev	5′-GATGCTGCTTACATGTCTCG-3′	60	40	269 bp
PSMC4-fw	5′-TGTTGGCAAAGGCGGTGGCA-3′			
PSMC4-rev	5′-TCTCTTGGTGGCGATGGCAT-3′	60	40	182 bp
DMPKex2-in2-fw	5´-GAGGGACGACTTCGAGATTCTGAA-3´			
DMPKex2-in2-rev	5´-CACCACGAGTCAAGTCAGGC-3´	67	40	92 bp

fw= forward; rev= reverse; bp= base pairs; Tm= melting temperature.

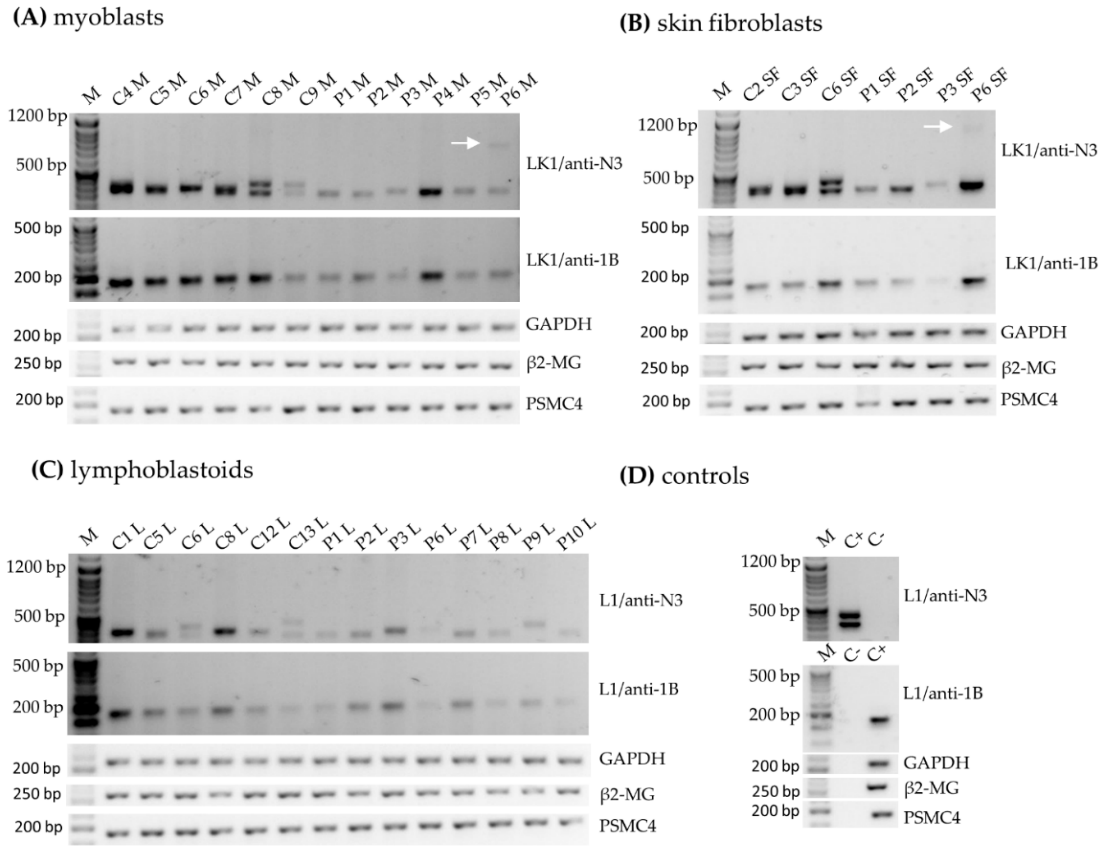

Figure S1. DM1-AS transcripts in DM1 cells of the additional two primer combinations studied. (**A**) DM1-AS specific primers LK1 and anti-N3/anti-1B for myoblasts (**A**), skin fibroblasts (**B**) and lymphoblastoids (**C**). RT-PCR controls are depicted in (**D**), C-= no DNA in RT-reaction and C+ = RNA of a control heart sample, the tissue used in original paper. Abbreviations: C= control; P= DM1 patient; bp= base pairs; M= marker. White arrows indicate the potential expanded repeat.

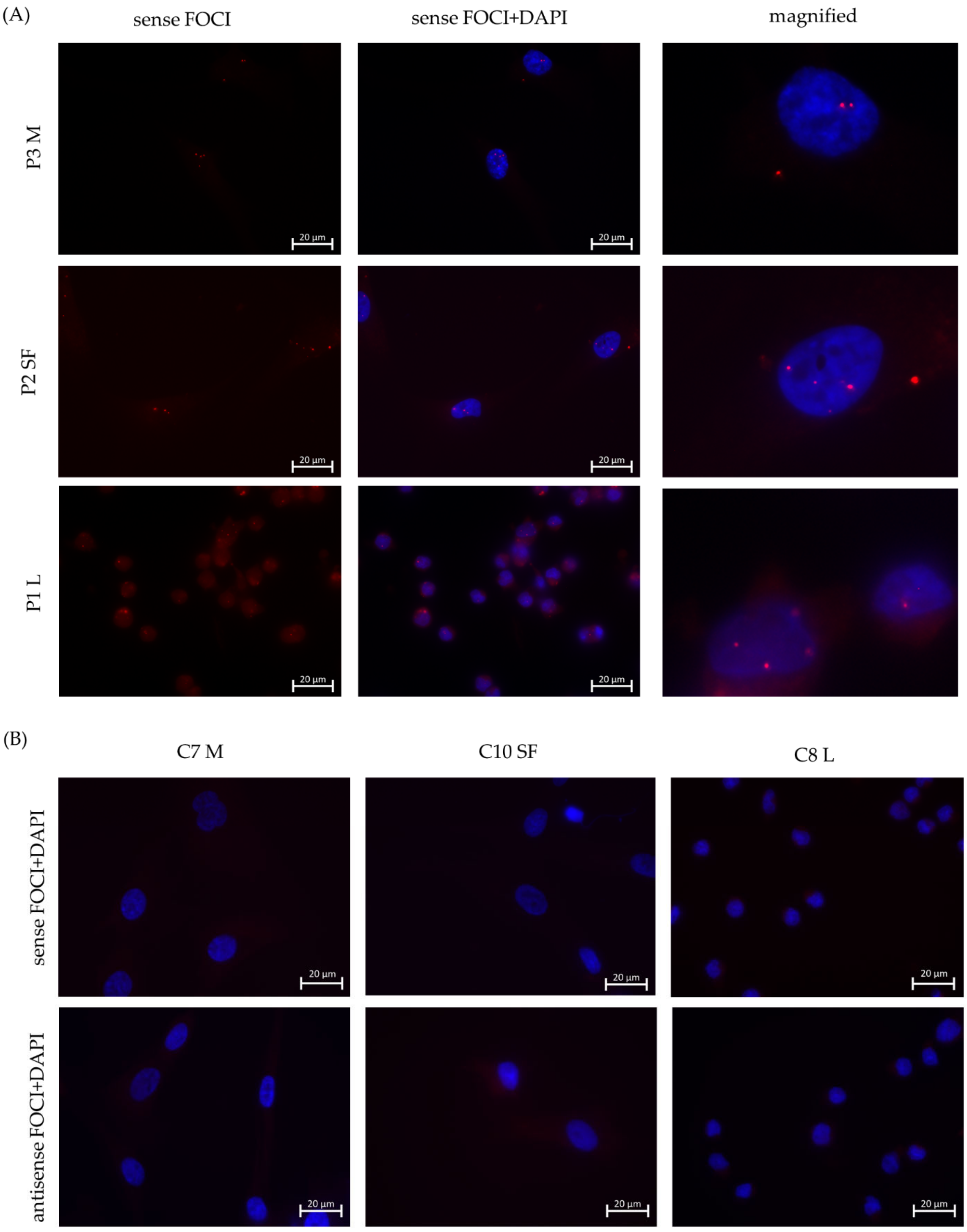

Figure S2. RNA foci overview. (A) sense RNA foci in all three cell types of DM1 patients. Cy3-labeled (CAG)10 probe (RED) showing sense RNA foci, nuclei indicated by DAPI (blue). Note that cytoplasmic sense RNA foci are present in both myoblasts and skin fibroblasts. (B) No presence of sense or antisense RNA foci in all three cell types of controls. Cy3-labeled (CAG)10 for sense RNA foci and Cy3-labeled (CTG)10 probe for antisense RNA foci (RED), nuclei indicated by DAPI (blue). P= DM1 patient; C= control; M= myoblasts; SF= skin fibroblasts; L= lymphoblastoids.

Table S2. Sense RNA foci details in DM1 myoblasts.

Myoblasts	Average RNA foci / cell		Maximum RNA foci / cell		Minimum RNA foci / cell		% Cells with RNA foci	
	nuclear	cytoplasm	nuclear	cytoplasm	nuclear	cytoplasm	nuclear	cytoplasm
P1	2.94 ± 1.70	0.00 ± 0.00	4.00	0.00	1.00	0.00	100.00	0.00
P3	4.30 ± 2.30	0.10 ± 0.31	11.00	1.00	1.00	0.00	100.00	10.00
P4	3.50 ± 1.40	0.50 ± 0.69	7.00	2.00	2.00	0.00	100.00	40.00
P5	3.00 ± 1.86	0.20 ± 0.42	8.00	1.00	1.00	0.00	100.00	20.00
Mean	3.44 ± 1.89	0.27 ± 0.52	7.50 ± 2.89	1.00 ± 0.82	1.25 ± 0.50	0.00 ± 0.00	100.00 ± 0.00	17.50 ± 17.08

Average/mean ± SD.

Table S3. Antisense RNA foci details in DM1 myoblasts.

Myoblasts	Average RNA foci / cell		Maximum RNA foci / cell		Minimum RNA foci / cell		% Cells with RNA foci	
	nuclear	cytoplasm	nuclear	cytoplasm	nuclear	cytoplasm	nuclear	cytoplasm
P1	0.30 ± 0.57	0.05 ± 0.22	2.00	1.00	0.00	0.00	25.00	5.00
P3	1.10 ± 0.97	0.70 ±1.30	3.00	4.00	0.00	0.00	70.00	30.00
P4	0.25 ± 0.72	0.15 ± 0.49	1.00	1.00	0.00	0.00	15.00	10.00
P5	0.35 ± 0.67	0.10 ± 0.45	2.00	2.00	0.00	0.00	25.00	5.00
Mean	0.50 ± 0.81	0.25 ± 0.77	2.00 ± 0.82	2.00 ± 1.41	0.00 ± 0.00	0.00 ± 0.00	33.75 ± 24.62	12.50 ± 11.90

Average/mean ± SD.

Table S4. Sense RNA foci details in DM1 skin fibroblasts.

Skin fibroblasts	Average RNA foci / cell		Maximum RNA foci / cell		Minimum RNA foci / cell		% Cells with RNA foci	
	nuclear	cytoplasm	nuclear	cytoplasm	nuclear	cytoplasm	nuclear	cytoplasm
P2	3.55 ±1.61	0.40 ± 0.82	8.00	2.00	1.00	0.00	100.00	25.00
P4	3.60 ± 1.43	0.10 ± 0.31	6.00	1.00	1.00	0.00	100.00	10.00
P7	3.45 ± 1.19	0.00 ± 0.00	6.00	0.00	0.00	0.00	100.00	0.00
P8	3.85 ± 1.63	0.35 ± 0.81	8.00	3.00	2.00	0.00	100.00	20.00
Mean	3.61 ± 1.45	0.21 ± 0.61	7.00 ± 1.17	1.50 ± 1.29	1.00 ± 0.82	0.00 ± 0.00	100.00 ± 0.00	13.75 ± 11.09

Average/mean ± SD.

Table S5. Antisense RNA foci details in skin fibroblasts.

Skin fibroblasts	Average RNA foci / cell		Maximum RNA foci / cell		Minimum RNA foci / cell		% Cells with RNA foci	
	nuclear	cytoplasm	nuclear	cytoplasm	nuclear	cytoplasm	nuclear	cytoplasm
P2	0.65 ± 0.93	0.05 ± 0.22	4.00	1.00	0.00	0.00	50.00	5.00
P4	0.20 ± 0.41	0.15 ± 0.49	1.00	2.00	0.00	0.00	20.00	10.00
P7	0.20 ± 0.41	0.10 ± 0.31	1.00	1.00	0.00	0.00	20.00	10.00
P8	0.20 ± 0.52	0.10 ± 0.31	2.00	1.00	0.00	0.00	15.00	10.00
Mean	0.31 ± 0.63	0.10 ± 0.34	2.00 ± 1.41	1.25 ± 0.50	0.00 ± 0.00	0.00 ± 0.00	26.25 ± 16.01	8.75 ± 2.50

Average/mean ± SD.

Table S6. Sense RNA foci details in lymphoblastoids.

Lymphoblastoids	Average RNA foci / cell		Maximum RNA foci / cell		Minimum RNA foci / cell		% Cells with RNA foci	
	nuclear	cytoplasm	nuclear	cytoplasm	nuclear	cytoplasm	nuclear	cytoplasm
P1	0.85 ± 1.09	N.D.	4.00	N.D.	0.00	N.D.	50.00	N.D.
P2	0.81 ± 1.11	N.D.	3.00	N.D.	0.00	N.D.	44.00	N.D.
P7	1.06 ± 1.30	N.D.	5.00	N.D.	0.00	N.D.	58.80	N.D.
P8	1.29 ± 1.38	N.D.	5.00	N.D.	0.00	N.D.	60.00	N.D.
Mean	1.01 ± 1.22	N.D.	4.25 ± 0.96	N.D.	0.00 ± 0.00	N.D.	53.20 ± 7.58	N.D.

Average/mean ± SD. Cytoplasm too small to make an accurate distinction, therefore cytoplasmic RNA foci were not counted, N.D. = not determined.

Table S7. Antisense RNA foci details in lymphoblastoids.

Lymphoblastoids	Average RNA foci / cell		Maximum RNA foci / cell		Minimum RNA foci / cell		% Cells with RNA foci	
	**nuclear**	**cytoplasm**	**nuclear**	**cytoplasm**	**nuclear**	**cytoplasm**	**nuclear**	**cytoplasm**
P1	0.05 ± 0.22	N.D.	1.00	N.D.	0.00	N.D.	5.00	N.D.
P2	0.15 ± 0.37	N.D.	1.00	N.D.	0.00	N.D.	15.00	N.D.
P7	0.00 ± 0.00	N.D.	0.00	N.D.	0.00	N.D.	0.00	N.D.
P8	0.00 ± 0.00	N.D.	0.00	N.D.	0.00	N.D.	0.00	N.D.
Mean	0.05 ± 0.22	N.D.	0.50 ± 0.58	N.D.	0.00 ± 0.00	N.D.	5.00 ± 7.07	N.D.

Average/mean ± SD. Cytoplasm too small to make an accurate distinction, therefore cytoplasmic RNA foci were not counted, N.D. = not determined.

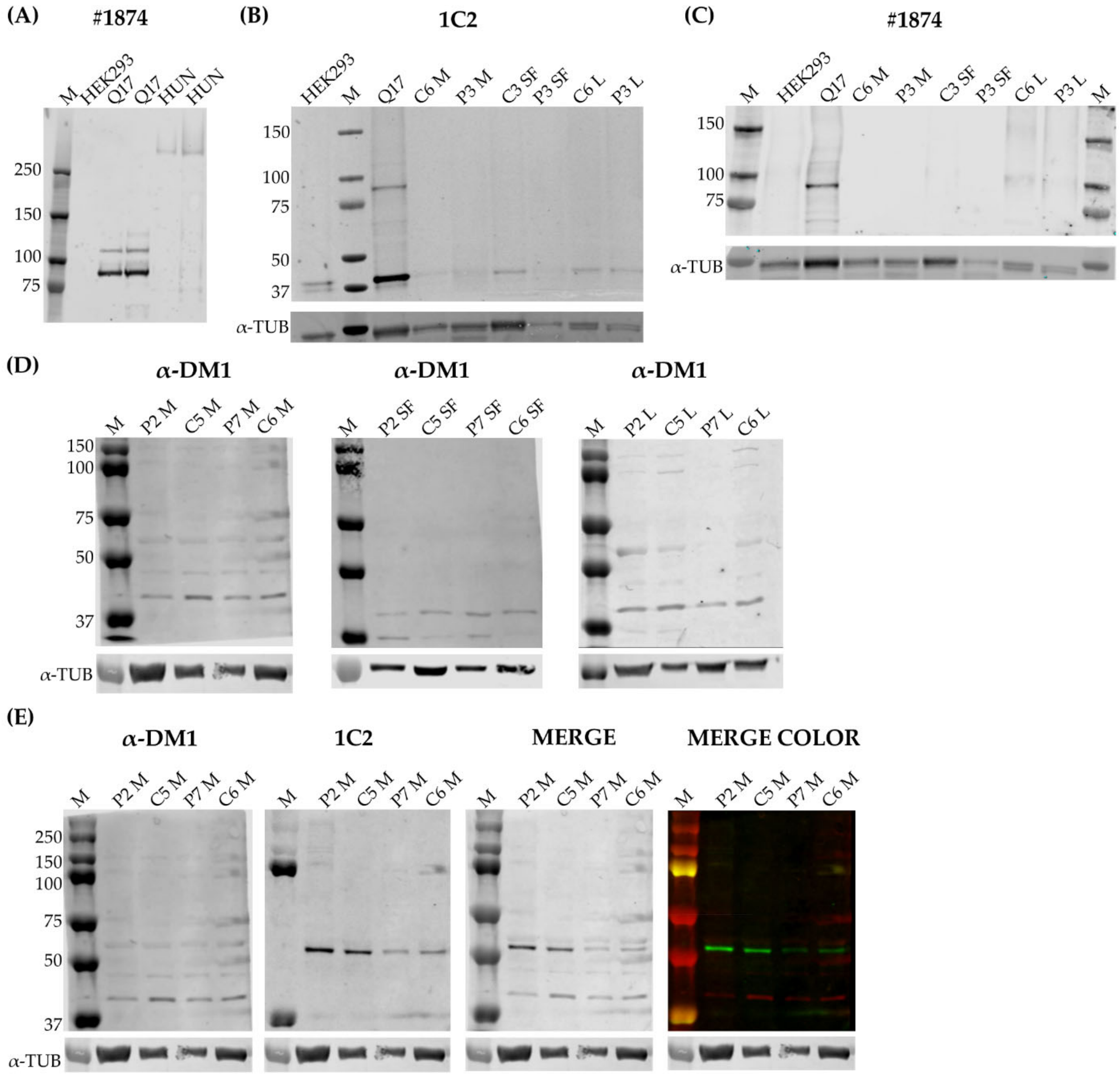

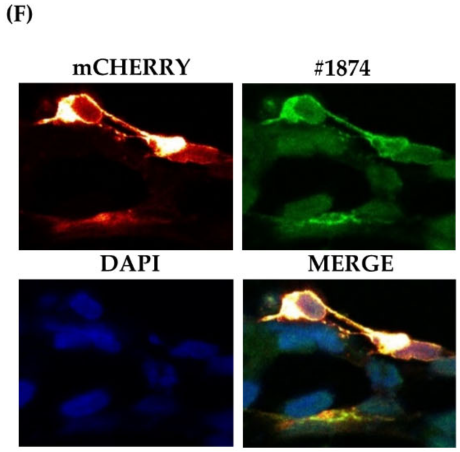

Figure S3. Quantitative analysis of the polyGln RAN protein and antibody validation. (**A**) Immunoblot showing the #1874 antibody recognizing the Q17 vector and the expanded Huntingtin protein in lymphoblastoid cells originating from a Huntington's patient. (**B**) a representative immunoblot with 1C2 1:1000, showing the exact same pattern (n = 4). (**C**) Immunoblot stripped and reprobed with #1874 after cutting off the 42 kD band. (**D**) Immunoblots with α-DM1 antibody 1:1000 for myoblasts, skin fibroblasts and lymphoblastoids. (**E**) Immunoblot with α-DM1 and 1C2 showing that both antibodies are not binding the same proteins. α-DM1 in red, 1C2 in green. (**F**) Immunofluorescence of HEK293 cells transfected with the Q17 vector. Transfection is indicated by mCherry (RED), PolyGln is indicated with #1874 (GREEN) and nuclei by DAPI (BLUE). Abbreviations: C = control; P = DM1 patient; M = myoblasts; SF = skin fibroblasts; L = lymphoblastoids; Q17 = Q17 vector; HUN = lymphoblastoids of a Huntington's patient.

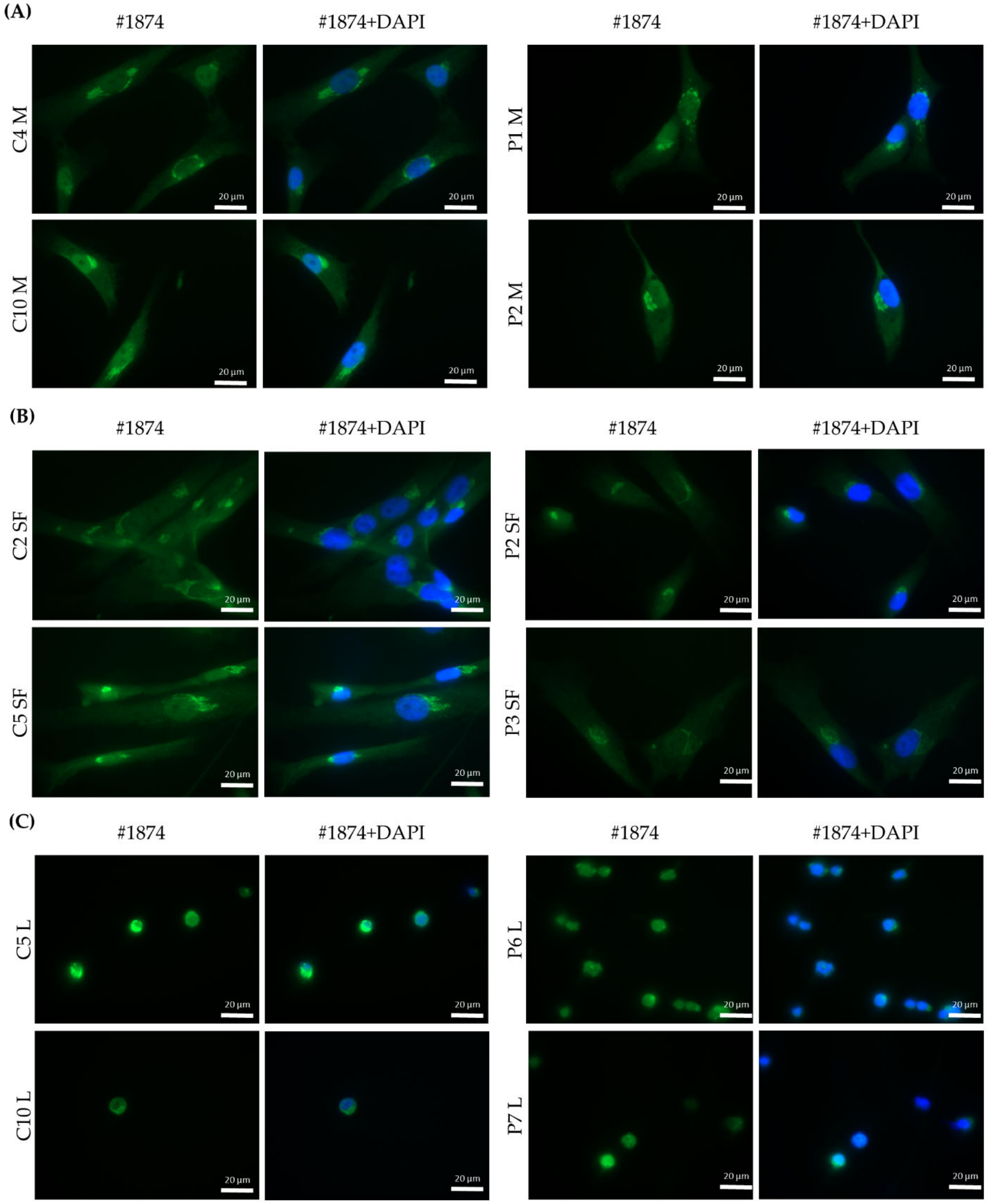

Figure S4. Qualitative analysis of polyGln RAN proteins with the #1874 antibody of additional samples. (**A**) Immunofluorescence polyGln staining with #1874 (alexa fluor-488, green) of human control and DM1 myoblasts (C: *n* = 5, P: *n* = 6). Nuclei indicated by DAPI (blue) (**B**) Immunofluorescence polyGln staining with #1874 (alexa fluor-488, green) of human control and DM1 skin fibroblasts (C: *n* = 8, P: *n* = 5). (**C**) Immunofluorescence polyGln staining with #1874 (alexa fluor-488, green) of human control and DM1 lymphoblastoids (C: *n* = 4, P: *n* = 5). Nuclei indicated by DAPI (blue). Abbreviations: C = control; P = DM1 patient; M = myoblasts; SF = skin fibroblasts; L = lymphoblastoids.

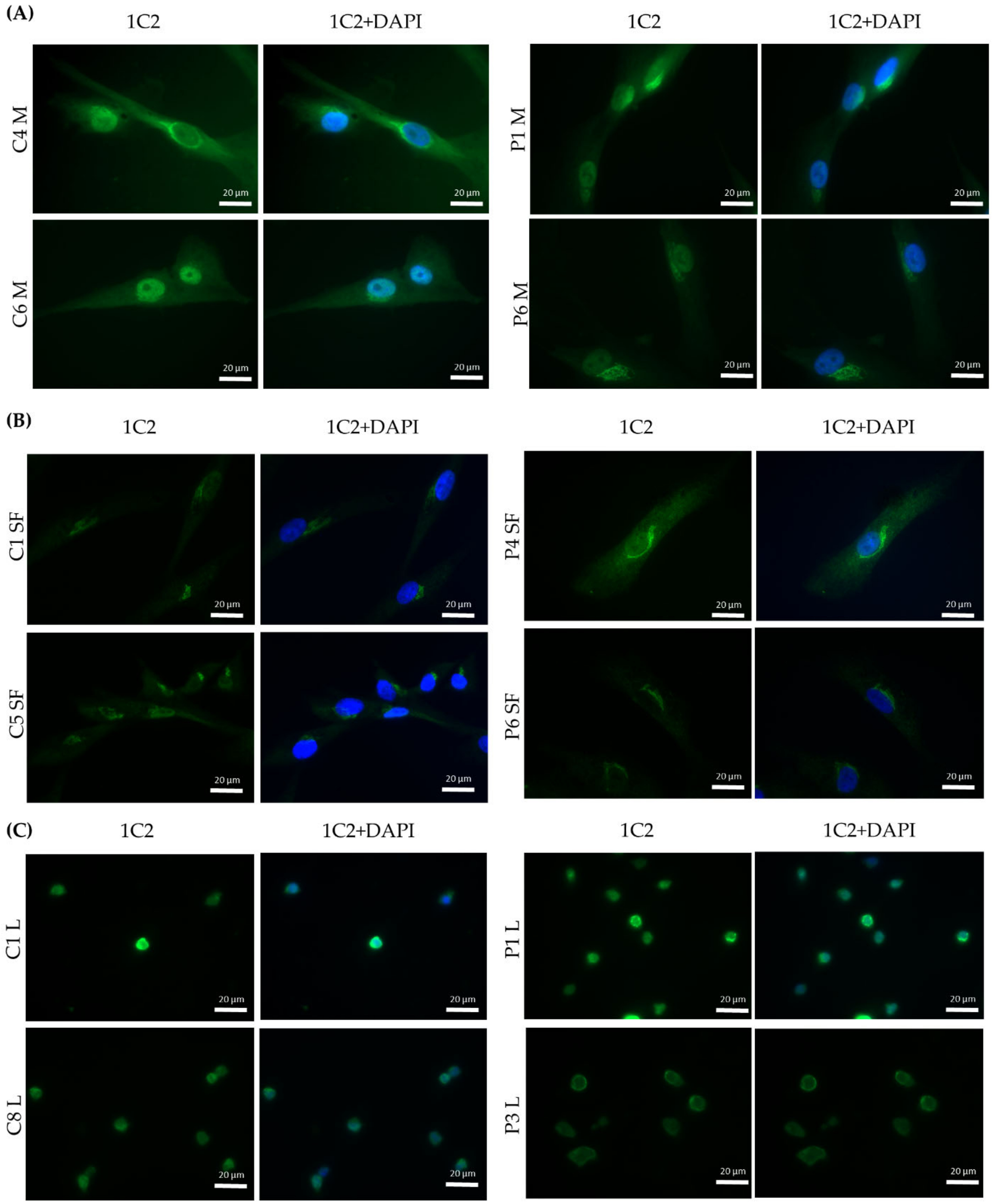

Figure S5. Qualitative analysis of polyGln RAN proteins with the 1C2 antibody. (A) Immunofluorescence polyGln staining with 1C2 (alexa fluor-488, green) of human control and DM1 myoblasts (C: n=6, P: n=6). Nuclei indicated by DAPI (blue) (B) Immunofluorescence polyGln staining with 1C2 (alexa fluor-488, green) of human control and DM1 skin fibroblasts (C: n=8, P: n=8). (C) Immunofluorescence polyGln staining with 1C2 (alexa fluor-488, green) of human control and DM1 lymphoblastoids (C: n=4, P: n=6). Nuclei indicated by DAPI (blue). Abbreviations: C= control; P= DM1 patient; M= myoblasts; SF= skin fibroblasts; L= lymphoblastoids.

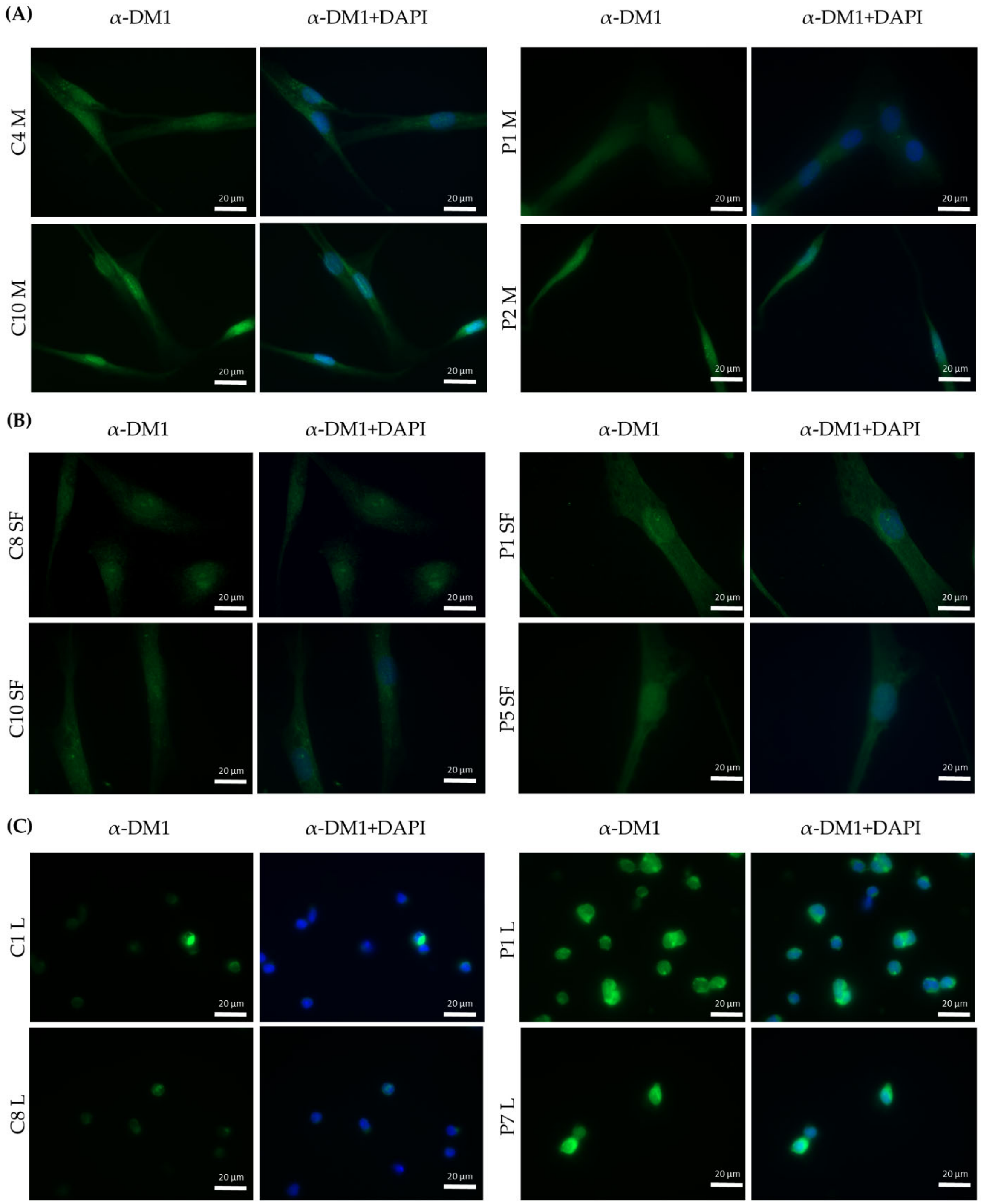

Figure S6. Qualitative analysis of polyGln RAN proteins with the α-DM1 antibody. (**A**) Immunofluorescence polyGln staining with α-DM1 (alexa fluor-488, green) of human control and DM1 myoblasts (C: n = 3, P: n = 4). Nuclei indicated by DAPI (blue) (**B**) Immunofluorescence polyGln staining with α-DM1 (alexa fluor-488, green) of human control and DM1 skin fibroblasts (C: n = 4, P: n = 4). (**C**) Immunofluorescence polyGln staining with α-DM1 (alexa fluor-488, green) of human control and DM1 lymphoblastoids (C: n = 4, P: n = 4). Nuclei indicated by DAPI (blue). Abbreviations: C = control; P = DM1 patient; M = myoblasts; SF = skin fibroblasts; L = lymphoblastoids.

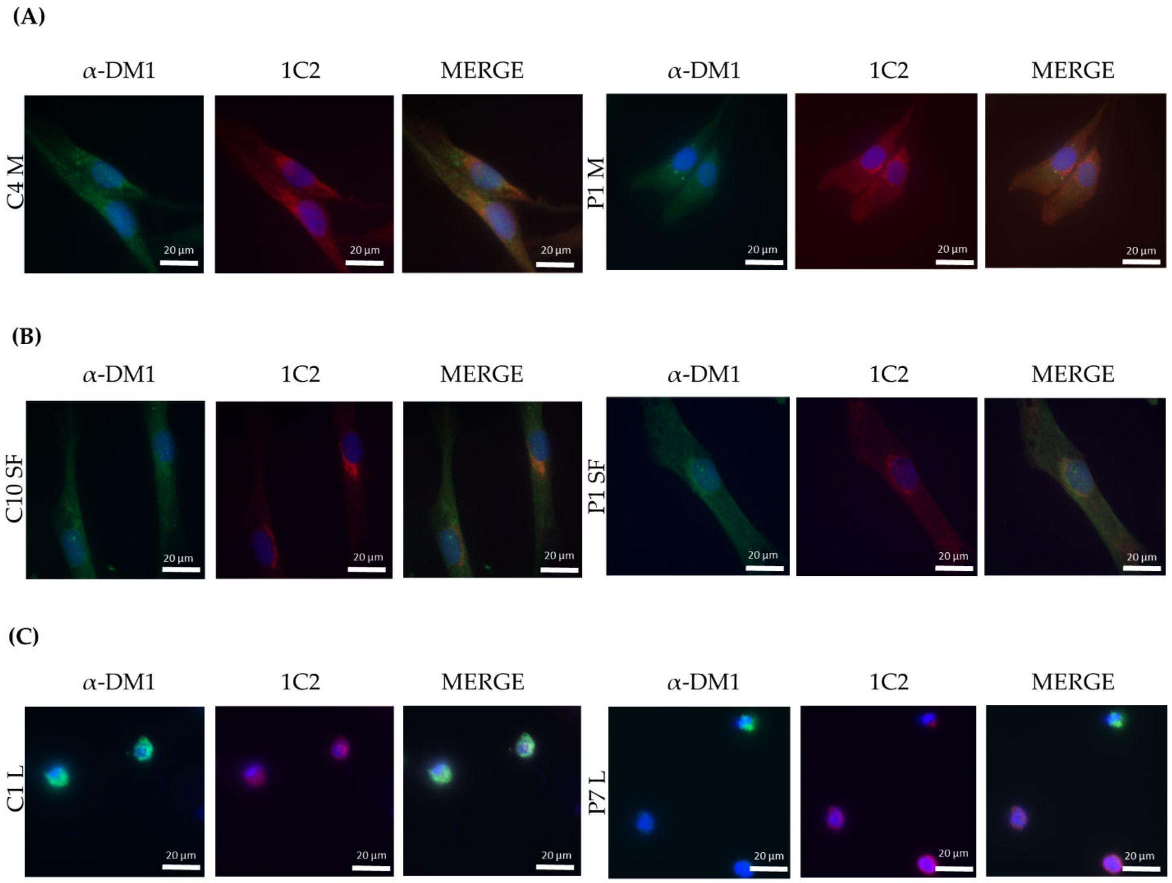

Figure S7. Double immunofluorescence with the α-DM1 antibody and 1C2 antibody. (**A**) Immunofluorescence polyGln staining with α-DM1 (alexa fluor-488, green) and 1C2 antibody (alexa fluor-594) of human control and DM1 myoblasts. Nuclei indicated by DAPI (blue) (**B**) Immunofluorescence polyGln staining with α-DM1 (alexa fluor-488, green) and 1C2 antibody (alexa fluor-594) of human control and DM1 skin fibroblasts (**C**) Immunofluorescence polyGln staining with α-DM1 (alexa fluor-488, green) and 1C2 antibody (alexa fluor-594) of human control and DM1 lymphoblastoids. Nuclei indicated by DAPI (blue). Abbreviations: C = control; P = DM1 patient; M = myoblasts; SF = skin fibroblasts; L = lymphoblastoids.
